# Opioid use and patient outcomes in an Australian hip and knee arthroplasty cohort

**DOI:** 10.1111/ans.17969

**Published:** 2022-08-08

**Authors:** Phil Huang, Jack Brownrigg, Justin Roe, David Carmody, Leo Pinczewski, Benjamin Gooden, Matthew Lyons, Lucy Salmon, Ka Martina, Joanna Crighton, Michael O'Sullivan

**Affiliations:** ^1^ North Sydney Orthopaedic Research Group Sydney New South Wales Australia; ^2^ Orthopaedic Services The Mater Hospital Sydney New South Wales Australia; ^3^ School of Medicine University of Notre Dame Sydney New South Wales Australia; ^4^ North Sydney Orthopaedic and Sports Medicine Centre Sydney New South Wales Australia; ^5^ School of Clinical Medicine, Faculty of Medicine and Health UNSW Sydney New South Wales Australia

**Keywords:** arthroplasty, hip, knee, opioids, pain control

## Abstract

**Background:**

To determine the prevalence of opioid use in Australian hip (THA) or knee (TKA) cohort, and its association with outcomes.

**Methods:**

About 837 primary THA or TKA subjects prospectively completed Oxford Scores, and Knee or Hip Osteoarthritis Outcomes Score(KOOS/HOOS) and opioid use in the previous week before arthroplasty. Subjects repeated the baseline survey at 6 months, with additional questions regarding satisfaction.

**Results:**

Opioid use was reported by 19% preoperatively and 7% at 6 months. Opioid use was 46% at 6 weeks and 10% at 6 months after TKR, and 16% at 6 weeks and 4% at 6 months after THR. Preoperative opioid use was associated with back pain(OR 2.2, *P* = 0.006), anxiety or depression(OR 1.8, *P* = 0.001) and Oxford knee scores <30(OR 5.6, *P* = 0.021) in TKA subjects, and females in THA subjects(OR 1.7, *P* = 0.04). There was no difference between preoperative opioid users and non‐users for satisfaction, or KOOS or HOOS scores at 6 months. 77% of patients taking opioids before surgery had ceased by 6 months, and 3% of preoperative non users reported opioid use at 6 months. Opioid use at 6 months was associated with preoperative use (OR 6.6–14.7, *P* < 0.001), and lower 6 month oxford scores (OR 4.4–83.6, *P* < 0.01).

**Conclusion:**

One in five used opioids before arthroplasty. Pre‐operative opioid use was the strongest risk factor for opioid use at 6 months, increasing odds 7–15 times. Prolonged opioid use was rarely observed in the opioid naïve (<5% TKA and 1% THA). Preoperative opioid use was not associated with inferior outcomes or satisfaction.

## Graphical abstract

In this Australian privately insured cohort opioid use was reported by 19% preoperatively and 7% at 6 months. Pre‐operative opioid use was the strongest risk factor for opioid use at 6 months, increasing the odds 7–15 times. Preoperative opioid use was not associated with inferior outcomes or satisfaction.

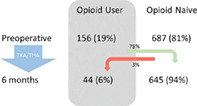

## Introduction

The ‘opioid epidemic’ is a worldwide affliction with 110 000 deaths attributed to opioid use in 2017, an increase by 77% over a decade.^1^ In the United States (US), the economic burden of opioid use disorder was calculated at $1.02 trillion in 2017,^2^ and an estimated 9.4 million people misused prescription opioids in 2019.^3^ In 2014–2016 Australia had the eighth highest opioid consumption in a comparison of 167 countries, and opioid related deaths have nearly doubled over the last 10 years.^4^ The use of pharmaceutical opioids is particularly high in the elderly, with 44% of opioid prescriptions dispensed in those aged 65 and more.^4^

Pain is a common and disabling feature of hip and knee osteoarthritis. Whilst opioids are commonly prescribed, their use for osteoarthritis is not recommended,^5^ and do not provide any benefit over ibuprofen or paracetamol to manage arthritic pain.^6^ Opioid use in the arthroplasty population in increasing, and has been associated with adverse outcomes with respect to length of hospital stay,^7^ patient reported outcomes,^8^ mortality and morbidity,^9^ revision surgery^10^ and infection,^11^ knee stiffness,^10^, ^11^ and prolonged post‐operative opioid use^7^, ^11^, ^12^, ^13^ compared to non‐opioid users.

There is considerable variation in rates of opioid use across arthroplasty populations, with ranges from 16% to 90% before, and 11%–41% >3 months after arthroplasty.^7^, ^8^, ^11^, ^13^, ^14^, ^15^, ^16^, ^17^, ^18^, ^19^, ^20^ Most of these epidemiological studies arise from populations in the US, where there are much higher rates of opioid use, compared to Australasia and Europe. The rate of opioid use in Australia has been reported in exclusively veteran and uninsured cohorts, and varies from 16% to 46% preoperatively, and 30%–36% >3 months after arthroplasty,^15^, ^16^, ^21^ with elevated rates observed in veterans. The aim of this study was to determine the prevalence of opioid use before and after THA and TKA in a privately insured Australian population and examine its association with patient factors and patient reported outcomes.

## Patients and methods

### Study design and participants

The prospective cohort study was performed at the North Sydney Orthopaedic and Sports Medicine Centre, Sydney, Australia. Eligible patients were undergoing primary elective THA or TKA under the care of one of seven experienced surgeons between May 2019 and October 2020 at a single hospital. The study was approved by St Vincent's Human Research Ethics Committee, Darlinghurstand all subjects provided informed consent. Subjects (*n* = 864) completed a series of patient reported outcomes measures (PROMs) preoperatively, at 6 weeks and 6 months after surgery. The routine data collection includes numerous demographic and operative variables. Subjects were excluded in the case of incomplete baseline PROMs (*n* = 23), death during the follow up (*n* = 1), and revision arthroplasty surgery (*n* = 3). The remaining 837 subjects formed the study group.

### Opioid use

Subjects were surveyed regarding their consumption of any opioids within the previous 7 days before surgery, at 6 weeks and at 6 months following surgery with sufficient detail to determine a daily morphine equivalent dose (MED). If opioid consumption was inconsistent then the maximum daily MED was used. Opioid use was defined as any opioid consumption within the previous 7 days for any indication. Opioid use was further categorized as opioid‐naïve (0 mg MED), opioid exposed (<60 mg MED) or opioid tolerant (≥60 mg MED).^22^

### 6 week review

At 6 weeks subjects reported average pain in the last 24 h on a visual analog scale (VAS), analgesia and opioid use. Complications within 6 weeks were documented.

### Patient reported outcome measures

Preoperative and 6 month PROMs included the disease specific short form HOOS or KOOS JR,^23^, ^24^ and Oxford Knee or Hip Score.^25^, ^26^ The EQ5D^27^ was selected as a widely used generic measure of health status, measuring mobility, self‐care, activity, pain and anxiety. Satisfaction was assessed after surgery with four additional questions: would they have the same surgery again under the same circumstances (Yes/No/Unsure), grading satisfaction with the results of surgery on 5‐point Likert scale from ‘very disappointed’ to ‘very satisfied’, and grading ‘overall, how the problems are now with your hip or knee on which you had surgery, compared to before you had your operation?’ (much better/a little better/about the same/a little worse/much worse). The preoperative and 6 month survey was completed online using REDCap electronic data capture tools,^28^ or using a paper copy if preferred.

### Statistical analysis

Data analysis was performed using SPSS data analysis software version 27. Frequencies were compared between groups using chi square tests, and means compared with t‐tests. Missing data was not imputed and complete case analysis was used for regression models. Opioid use was dichotomised as any or none for the regression model. Univariate logistic regression analysis of opioid use was analysed for TKA and THA groups separately for the factors of gender, age <55, obesity (BMI 30 or more), any anxiety or depression on EQ5D, discharge destination (home or inpatient rehabilitation), Oxford score <30, back pain and preoperative opioid use. Significant factors were entered into multivariate logistic model in a forward fashion. Odds Ratios for each factor with 95% confidence intervals and statistical p value was determined.

## Results

**Fig. 1:**
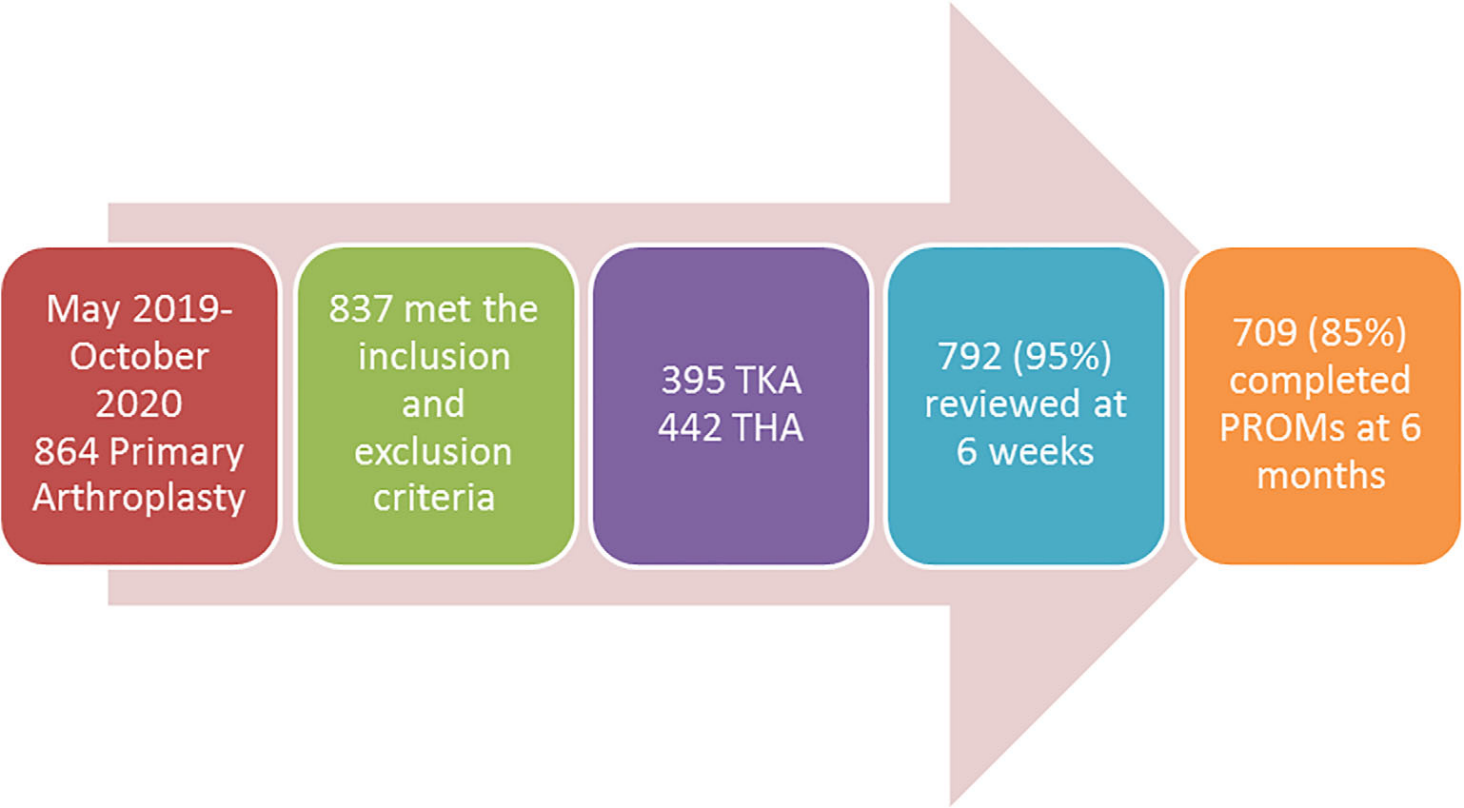
Participant flow.

About 837 subjects underwent primary THA or TKA consented to participate and completed preoperative PROMs. Of these, 792 completed PROMS at 6 weeks and 709 completed PROMs at 6 months (Fig. 1). The demographics and operative measures for the TKA and THA cohort is shown in Table 1.

**Table 1:** Demographics and operative and baseline measures for the THA and TKA subjects

	Knee arthroplasty (*n* = 395)	Hip arthroplasty (*n* = 442)	*P*
Females	198 (50%)	238 (54%)	0.282
Mean age	69.1	67.5	0.017
ASA grade 1 or 2	229 (58%)	359 (81%)	0.001
Obesity (BMI 30 or more)	195 (49%)	146 (33%)	0.001
Diagnosis osteoarthritis	368 (93%)	390 (88%)	0.034
Any depression or anxiety	198 (50%)	218 (49%)	0.816
Back Pain VAS 4 or more	135 (34%)	204 (46%)	0.001
Preoperative Oxford Score < 30	315 (80%)	341 (77%)	0.362
Mean length of stay (days)	5.2	4.6	0.001
Discharge to home	156 (48%)	225 (55%)	0.177

### Opioid use in THA and TKA cohorts

**Fig. 2:**
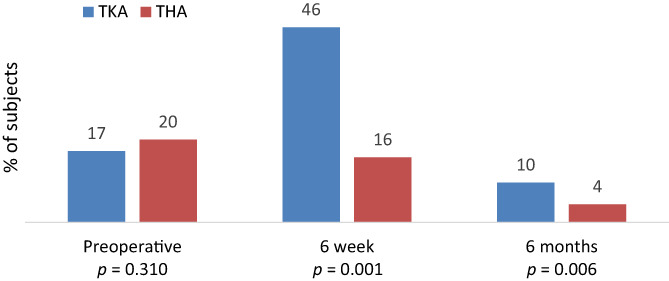
Incidence of any opioid use in TKA and THA subjects at each review.

Opioid use was reported by 154 (18%) before surgery, 236 (28%) at 6 weeks and 48 (7%) at 6 months. The prevalence of opioid use at each review is shown for TKA and THA subjects in Figure 2. The prevalence of opioid use was equivalent between THA and TKA preoperatively, but three times higher in the TKA subjects at 6 weeks and 2.5× higher in the TKA subjects at 6 months. Opioid use was recorded with insufficient detail to determine MED in 17 subjects before surgery and in six subjects at 6 weeks. The mean MED in opioid users was 24.7 mg (range 1–263) preoperatively, 23.7 mg (range 1.3–203) at 6 weeks and 20.0 mg (range 2–111) at 6 months. There was no significant difference in mean MED between THA and TKA subjects preoperatively (*P* = 0.933), at 6 weeks(*P* = 0.402), or 6 months (*P* = 0.999). The number of subjects taking opioids graded according to MED dose for THA and TKA subjects is shown in Table 2.

**Table 2:** Opioid use in TKA and THA subjects graded by morphine equivalent daily dose

	Knee arthroplasty	Hip arthroplasty	*P*
Preoperative opioid (*n*)	389	431	
Naïve (MED = 0 mg)	328 (84%)	354 (82%)	0.474
Exposed (MED < 60 mg)	54 (14%)	64 (15%)	
Tolerant (MED ≥ 60 mg)	7 (2%)	13 (3%)	
6 week (*n*)	362	425	
Naïve (MED = 0 mg)	196 (54%)	361 (85%)	0.001
Exposed (MED < 60 mg)	151 (42%)	59 (14%)	
Tolerant (MED ≥ 60 mg)	15 (4%)	5 (1%)	
6 month (*n*)	338	371	
Naïve (MED = 0 mg)	306 (90%)	355 (96%)	0.012
Exposed (MED < 60 mg)	29 (9%)	16 (4%)	
Tolerant (MED ≥ 60 mg)	3 (1%)	0 (0%)	

### Opioid user to non‐user

**Fig. 3:**
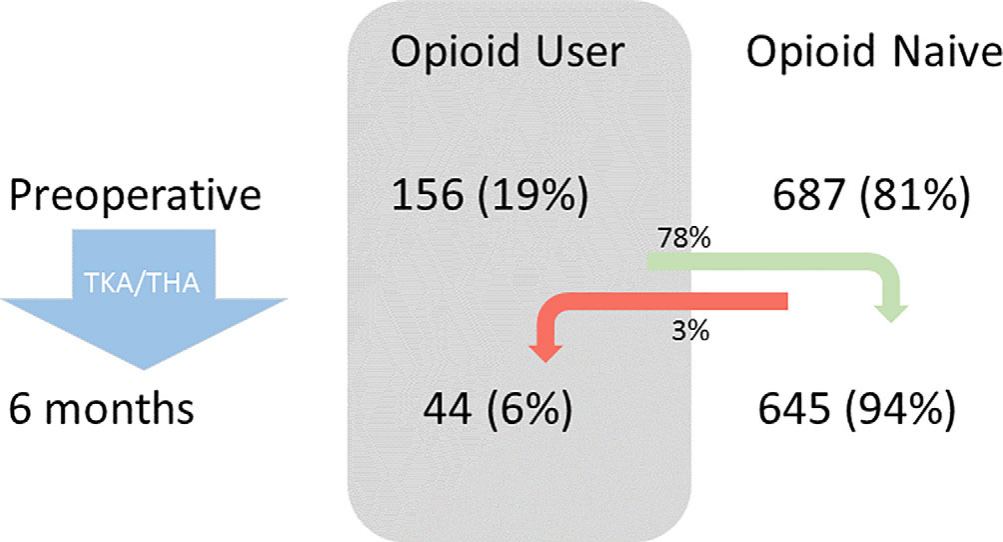
Change in incidence of any opioid use from preoperative to 6 months review.

The change in opioid use is shown in Figure 3. In the TKA cohort 67 (17%) were taking opioids preoperatively, of these 68% had ceased opioids at 6 months. Of the 328 opioid naïve TKA subjects, 5% reported opioid use at 6 months. In the THA cohort 87 (20%) were taking opioids preoperatively and 83% had ceased opioids at 6 months. Of the 355 opioid naïve THA subjects, 1% reported opioid use at 6 months.

### Regression analysis of opioid use

The logistic multivariate regression analysis of opioid use is shown for TKA subjects and THA subjects in Tables 3, 4, 5.

**Table 3:** Multivariate logistic regression of preoperative opioid use

	Total knee arthroplasty					Total hip arthroplasty				
Preoperative opioid use	No opioid use *N* = 328	Any opioid use *N* = 67	OR	95%CI	*P*	No opioid use *N* = 355	Any opioid use *N* = 87	OR	95%CI	*P*
Female	158 (48%)	40 (60%)	1.2	0.7–2.2	0.447	183 (52%)	55 (63%)	1.7	1.0–2.8	0.041**
Obesity (BMI 30 or more)	153 (47%)	42 (67%)	1.7	0.3–3.0	0.083	110 (31%)	36 (41%)	1.5	0.9–2.6	0.095
Back Pain VAS 4 or more	97 (30%)	38 (58%)	2.2	1.3–3.9	0.006**	160 (46%)	44 (51%)	1.0	0.6–1.6	0.918
Anxiety or Depression	147 (45%)	51 (76%)	2.8	1.5–5.2	0.001**	163 (46%)	55 (63%)	2.0	0.9–4.1	0.075
Preoperative Oxford Score < 30	250 (76%)	65 (98%)	5.6	1.3–24.0	0.021**	264 (74%)	77 (89%)	2.1	0.9–5.0	0.088

Abbreviations: CI, confidence interval; OR, odds ratio.

** Denotes statistically significant factors.

**Table 4:** Multivariate logistic regression of 6 week opioid use

	Total knee arthroplasty					Total hip arthroplasty				
6 week opioid use	No opioid use *N* = 196	Any opioid use *N* = 170	OR	95%CI	*P*	No opioid use *N* = 361	Any opioid use *N* = 66	OR	95%CI	*P*
Preoperative opioid use	20 (10%)	40 (24%)	3.5	1.6–7.5	0.001**	53 (15%)	28 (42%)	14.7	4.2–50.7	0.001**
Joint pain at 6 weeks VAS 5 or more	54 (28%)	66 (39%)	4.0	2.0–7.9	0.001**	160 (45%)	38 (59%)	8.5	2.9–25.0	0.001**
Attended inpatient rehabilitation	79 (45%)	85 (59%)	1.9	1.2–3.2	0.013**	139 (40%)	43 (68%)	2.4	1.3–4.5	0.008**
Preoperative back pain VAS 4 or more	54 (28%)	66 (39%)	1.1	0.6–1.8	0.860	160 (45%)	38 (59%)	1.5	0.8–2.9	0.205
Preoperative anxiety or depression	93 (47%)	91 (54%)	1.1	0.4–1.3	0.298	165 (46%)	42 (64%)	1.4	0.7–2.7	0.322
Preoperative Oxford Score < 30	142 (72%)	147 (87%)	1.3	0.7–2.5	0.411	270 (75%)	57 (86%)	1.1	0.5–2.6	0.870

Abbreviations: CI, confidence interval; OR, odds ratio.

*Note*: * Female gender and preoperative obesity not included in multivariate model as factor did not reach significance on univariate analysis.

** Denotes statistically significant factors.

**Table 5:** Multivariate logistic regression of 6 month opioid use

	Total knee arthroplasty					Total hip arthroplasty				
6 month opioid use	No opioid use *N* = 306	Any opioid use *N* = 32	OR	95%CI	*P*	No opioid use *N* = 355	Any opioid use *N* = 16	OR	95%CI	*P*
Preoperative opioid use	36 (12%)	17 (53%)	6.6	2.7–16.1	0.001**	60 (17%)	12 (75%)	14.7	4.2–50.7	0.001*
6 month Oxford Score < 30	17 (6%)	8 (25%)	4.4	1.4–13.8	0.010**	13 (4%)	5 (31%)	8.6	2.0–36.2	0.003*
Preoperative back pain VAS 4 or more	98 (32%)	17 (57%)	1.3	0.5–3.2	0.505	163 (46%)	11 (69%)	2.1	0.6–7.3	0.234
Preoperative anxiety or depression	142 (46%)	25 (78%)	1.9	0.7–5.0	0.203	172 (49%)	10 (63%)	1.4	0.4–4.8	0.625
Preoperative Oxford Score < 30	239 (78%)	30 (94%)	1.4	0.3–6.5	0.706	272 (77%)	13 (81%)	0.4	0.1–1.9	0.245

Abbreviations: CI, confidence interval; OR, odds ratio.

* Female gender and preoperative obesity not included in multivariate model as factor did not reach significance on univariate analysis.

** Denotes statistically significant factors.

### Outcomes by baseline opioid use

Preoperative TKA opioid users had a lower preoperative mean KOOS Score (39 versus 51, *P* = 0.001), Oxford Score (17 versus 24, *P* = 0.001), and EQ5D General Health Score (60 versus 68, *P* = 0.004), than non‐users. Preoperative THA opioid users had a lower preoperative mean HOOS Score (48 versus 57, *P* = 0.001), Oxford Score (18 versus 24, *P* = 0.001), and EQ5D General Health Score (57 versus 68, *P* = 0.001), than non‐users. There was no difference in mean acute length of stay between opioid users and non‐users for TKA (5.3 versus 5.2, *P* = 0.83) or THA (4.5 versus 4.7, *P* = 0.14). There was no difference in prevalence of readmission within 30 days between opioid users and non‐users for TKA (2% versus 2% *P* = 0.939) or THA (1% versus 2%, *P* = 0.753). Six months outcomes are compared between preoperative opioid users and opioid naïve for TKA in Table 6 and THA in Table 7.

**Table 6:** 6 month patient reported outcomes for preoperative opioid non user and opioid users in knee arthroplasty participants

6 month PROMS	Preoperative opioid non user *N* = 285	Preoperative opioid user *N* = 53	Cohens *d*	*P*
*N*	285	53		
Opioid use at 6 months	15 (5%)	17 (32%)		0.001*
KOOS score, mean	78.7	77.2	−0.20	0.482
KOOS score, mean ∆	27.7	40.1	−0.71	0.001*
Oxford score, mean	40.2	39.2	0.14	0.362
Oxford score, mean ∆	15.9	23.1	−0.86	0.001*
Any depression or anxiety (%)	52 (18%)	17 (32%)		0.022*
Any problems with mobility (%)	105 (37%)	27 (51%)		0.053
EQ5D General Health Score, mean	79.2	71.5	0.40	0.013*
Satisfied with surgery	264 (93%)	46 (89%)		0.308
Same surgery again	255 (90%)	46 (89%)		0.132
Better after surgery	272 (95%)	50 (96%)		0.303

* Denotes statistical significant difference between groups.

**Table 7:** 6 month patient reported outcomes for preoperative opioid non user and opioid users in hip arthroplasty participants

6 month PROMS	Preoperative opioid non user *N* = 299	Preoperative opioid user *N* = 72	Cohens d	*P*
Opioid use at 6 months	4 (1%)	12 (17%)		0.001*
HOOS score, mean	87.2	86.5	0.05	0.716
HOOS score, mean ∆	30.4	38.2	−0.48	0.001*
Oxford score, mean	43.0	41.3	0.27	0.047
Oxford score, mean ∆	18.6	23.3	−0.52	0.001*
Any depression or anxiety (%)	49 (16%)	20 (28%)		0.026*
Any problems with mobility (%)	87 (29%)	29 (40%)		0.066
EQ5D General Health Score, mean	77.5	78.8	−0.06	0.659
Satisfied with surgery	286 (97%)	69 (97%)		0.811
Same surgery again	282 (95%)	66 (94%)		0.531
Better after surgery	288 (97%)	69 (99%)		0.403

* Denotes statistical significant difference between groups.

## Discussion

In this Australian cohort 19% reported opioid use before arthroplasty. After TKA opioid use was 46% at 6 weeks and 10% at 6 months, and after THA opioid use was 16% at 6 weeks and 4% at 6 months. Preoperative opioid use did not diminish the post‐operative improvement nor the absolute pain and function scores, or rates of satisfaction. Preoperative opioid use in TKA subjects was associated with back pain, anxiety or depression and low Oxford knee scores. Preoperative opioid use in THA subjects was associated with females. Of those taking opioids before surgery 77% had ceased at 6 months after arthroplasty, and only 3% of opioid naïve subjects reported opioid use at 6 months. Opioid use at 6 months was associated with preoperative opioid use, and lower 6 month Oxford scores for both THA and TKA cohorts.

This study is the largest to assess the relationship between opioid use and PROMS in the last decade, the first in a cohort outside the US, and the first to include measures of satisfaction for THA and TKA subjects. When compared to non‐opioid users, those taking opioids before surgery had lower baseline disease specific, general health and depression/anxiety scores. Opioid users experienced a significantly greater improvement after surgery, compared to opioid naïve subjects so that their pain and function outcomes were equivalent at 6 months, as were their rates of satisfaction. This contrasts with other studies that have reported lower levels of improvement in pain score in opioid users compared to opioid naïve subjects.^8^, ^10^ Our cohort had considerably lower proportion taking >60 mg MEDD and lower mean MED doses than other studies by more than 50%,^10^, ^29^ which may explain the differences observed. This is supported by reports that opioid users who weaned opioid use by 50% experienced similar improvements to their opioid naïve counterparts.^29^ A recent systematic review and meta‐analysis involving 7356 patients concluded that opioid users had worse absolute PROM scores, but similar relative change in scores when compared to opioid naïve patients.^13^ We observed a greater improvement in opioid users, resulting in equivalent PROM between opioid users and non‐users at 6 months. If opioid use prior to arthroplasty is of a low dosage then at least equivalent PROMS, and rates of satisfaction to non‐users may be achieved.

Pre‐operative opioid use has an established effect on sustained post‐operative opioid use,^7^, ^12^, ^19^, ^20^, ^30^ and our study further supports this finding. Pre‐operative opioid use was the strongest risk factor for opioid consumption at 6 months, increasing the odds by 7–15 times for TKA and THA respectively, with a greater influence than poor Oxford Scores. A recent systematic review reported post‐operative opioid use to be 35%–68% in THA and TKA patients who took opioids pre‐operatively and 0.6%–4% in patients who were opioid naïve.^30^ In our study preoperative opioid users continued use at 6 months in 15%–31% of THA and TKA subjects respectively, compared to preoperative non users who used opioids at 6 months in only 6% of TKA and 1% of THA subjects. To minimize prolonged opioid use most effectively clinicians need to address it before arthroplasty, weaning to the lowest possible dose.^22^ As opioids are prescribed by internal medicine or primary care providers in 60%–70% of arthroplasty cases,^31^ careful coordination and communication between orthopaedic surgeons, patients and other primary care providers as to expectations for opioid use may be paramount to successfully managing prolonged opioid use in this population.^21^, ^31^

Opioid use before and after arthroplasty observed in this study were considerably lower than many previous studies, but considerable geographical variation is commonly reported.^19^ In the largest series of a US cohort from 2010 to 2014 Jin^19^ examined 473 781 subjects and found 31% of TKR and 41% of THR subjects reported opioid use in the month before arthroplasty. Outside the US relatively consistent rates of opioid use of 22%–28% preoperatively and 13%–16% at 3–6 months after surgery has been reported in New Zealand,^20^ and Scandinavia.^17^, ^18^ The lowest rates were seen in Finland with preoperative opioid use of 9%–14% in TKA and THA patients respectively, and 7% at 9–12 months after surgery,^32^ which is identical to the 7% we observed at 6 months. Higher opioid use based on prescription data in Australian veterans was 39%–46%, decreasing to 30% of THA and 36% of TKA at >3 months.^15^, ^16^ These rates are more than double the rate of our cohort, and similar to US based studies, but may be influenced by the exclusively veteran sample. Naylor^21^ examined 521 THA or TKA subjects in an Australian public hospital cohort of whom 16% reported regular opioid use before, and 30% at 3 months after arthroplasty. In our cohort opioid use within the previous week was reported by 18% before surgery, 28% at 6 weeks and only 7% at 6 months. Differences in opioid use may be influenced by the healthcare setting.^12^ Our subjects were all privately insured, a group who are likely to undergo surgery at an earlier stage of disease progression and therefore may have a shorter history of severe pain leading to opioid use, which may explain the variation. However observed differences may also be related to our shorter reporting window of 7 days rather than 30–90 days, or other inconsistencies in the definition of opioid use, whether it was determined from prescription data, dispensing records or patient reported recall, methods which all arguably have inherent flaws. These inconsistencies hamper comparisons universally, and have been recently addressed by a consensus group who recommend standardized definitions.^22^ Regretfully, our study was designed prior to publication of this recommendation so included questions regarding the previous 7 days rather than the 90 days suggested by the group. However it has been shown that opioid consumption is highest in the month prior to surgery,^19^, ^33^ which is when our baseline measure was performed. Regardless we remain encouraged by the very low rate of only 7% that continued opioid use, and <1% that would be categorized as opioid tolerant beyond 6 months.

Discharge to an inpatient rehabilitation centre compared to home was associated with greater odds of opioid use at 6 weeks by a factor of 2×, even when controlled for pain scores and preoperative opioid use, and this is supported by others.^33^ By international standards Australians have a high rate of inpatient rehabilitation use, in our cohort 51% of TKR and 45% of THR subjects attended inpatient rehabilitation. Over the past 5 years these rates are steadily decreasing, driven largely by the pressure from health insurers and lack of evidence. Protocols have been common in Australian inpatient rehabilitation units encouraging routine opioid administration pre‐emptively prior to rehabilitation. A consistent message across all health providers that opioids should only be considered in the acute post‐operative phase where possible, and in only presence of severe pain, rather than an appropriate preventative measure for longer term postoperative discomfort is warranted.

This study demonstrated a >2.5× higher prevalence of opioid use in the TKA subjects compared to the THA subjects, despite equivalent pre‐operative use. A similar trend was observed in New Zealand^33^ and Finish^32^ arthroplasty cohorts. It is generally accepted that TKA is a more painful procedure than THA which is reflected in higher prevalence of opioid use.

This study has strengths and limitations. The cohorts were recruited from a private hospital in metropolitan Sydney, so our findings may not be generalizable to uninsured, or other regions or countries. Attempts to reduce opioid consumption broadly across all communities have escalated considerably over recent years, so it is important to use current data to assess evolving changes in opioid management. Our definition of opioid consumption in terms of MED had a lower threshold than many other studies, but our period of assessment being within last 7 days may conversely be less strict criteria. We support the recent suggestion standardized definition proposed by a recent consensus group^22^ to enable more accurate comparisons. We relied on self‐reported accuracy for opioid use, which has flaws, as arguably all methods are.

This study demonstrated one in five Australians used opioids before arthroplasty. Pre‐operative opioid use was the strongest risk factor for opioid use at 6 months, increasing the odds by 7–15 times. Importantly, prolonged opioid use was rarely observed in the opioid naïve cohort with less than 5% of TKA and 1% of THA subjects, and persisting opioid tolerant users represented <1% after arthroplasty. Preoperative opioid use was not associated with inferior PROMS or satisfaction with arthroplasty.

## Author contributions

**Phil Huang:** Conceptualization; data curation; methodology; writing – original draft; writing – review and editing. **Jack Brownrigg:** Data curation; investigation; project administration; writing – review and editing. **Justin Roe:** Conceptualization; data curation; funding acquisition; methodology; writing – review and editing. **David Carmody:** Conceptualization; data curation; methodology; writing – review and editing. **Leo Pinczewski:** Conceptualization; data curation; methodology; writing – review and editing. **Benjamin Gooden:** Data curation; investigation; methodology; writing – review and editing. **Matthew Lyons:** Conceptualization; data curation; methodology; writing – review and editing. **Lucy Salmon:** Data curation; formal analysis; funding acquisition; methodology; writing – original draft; writing – review and editing. **Ka Martina:** Data curation; methodology; project administration; writing – review and editing. **Joanna Crighton:** Data curation; methodology; project administration; writing – review and editing. **Michael O'Sullivan:** Conceptualization; investigation; methodology; writing – review and editing.

## Conflict of interest

Phil Huang, Jack Brownrigg, Lucy J. Salmon, Ka Martina, Joanna Crigton, David Carmody, Benjamin Gooden have no relevant disclosures. Justin Roe is a paid consultant to Smith and Nephew and Pureplay Orthopaedics, holds shares in 360 Knee Systems, and receive research funding from Friends of the Mater and Corin. Leo Pinczewski holds stock and receives royalties from Australian Biotechnologies, and receives research support from Smith and Nephew. Matthew Christopher Lyons receive royalties from Depuy Synthes, is a paid consultant to Depuy Synthes, Corin, Zimmer Biomet, holds stock in NavBit and receives research support from Depuy Synthes. Michael O'Sullivan receives research support from Friends of the Mater and Corin.
